# Imaging atmospheric aerosol particles from a UAV with digital holography

**DOI:** 10.1038/s41598-020-72411-x

**Published:** 2020-09-30

**Authors:** Osku Kemppinen, Jesse C. Laning, Ryan D. Mersmann, Gorden Videen, Matthew J. Berg

**Affiliations:** 1 Earth System Science Interdisciplinary Center, University of Maryland, College Park, MD 20742 USA; 2 Department of Physics, University of Central Florida, Orlando, FL 32816-2385 USA; 3 Department of Physics, Kansas State University, Manhattan, KS 66506-2601 USA; 4 US Army Research Laboratory, 2800 Powder Mill Road, Adelphi, MD 20783-1197 USA

**Keywords:** Atmospheric science, Applied optics

## Abstract

The lack of quantitative characterization of aerosol particles and their loading in the atmosphere is one of the greatest uncertainties in climate-change science. Improved instrumentation capable of determining the size and shape of aerosol particles is needed in efforts to reduce this uncertainty. We describe a new instrument carried by an unmanned aerial vehicle (UAV) that images free-floating aerosol particles in the atmosphere. Using digital holography, the instrument obtains the images in a non-contact manner, resolving particles larger than ten micrometers in size in a sensing volume of approximately three cubic centimeters. The instrument, called the holographic aerosol particle imager (HAPI), has the unique ability to image multiple particles freely entering its sensing volume from any direction via a single measurement. The construction of HAPI consists of 3D printed polymer structures that enable a sufficiently low size and weight that it may be flown on a commercial-grade UAV. Examples from field trials of HAPI show images of freshly emitted tree pollen and mineral dust.

## Introduction

Perhaps the most consequential aspect of atmospheric aerosols is their effect on the Earth’s energy budget via radiative forcing through sunlight absorption and scattering^1–6^. Aerosol particles impact human health as well and serve as ice nuclei^7–9^ and cloud condensation nuclei^10–13^ thus influencing the hydrological cycle^14–16^. Studies find that the estimated radiative forcing is comparable to all other forcing factors, including greenhouse gases^5,6,16–19^. Yet, the uncertainty in the forcing value remains nearly as large as the value itself^19^, meaning the effect of aerosols on climate is the least understood^19–21^. The reason for this is, in part, due to the use of unrealistically simple particle-shape proxies in light scattering models for the forcing inputs to general circulation models^18–25^. Limited data is available for the *true* morphology of many particles, especially in the coarse mode aerosol (CMA) size range, i.e. particles nominally $>1 \mathrm{\mu m}$ in size, although some microscopy has been done, see^26,27^. As such, distributions of spheres or spheroids are often used in conjunction with ground and satellite data in simulations of the forcing^25,28–31^ although more sophisticated treatments are also attempted^32,33^. What remains needed are more accurate observations of aerosols, particularly their microphysical characteristics, from in situ measurements in the atmosphere^3,6,34,35^.

CMAs, such as airborne mineral dust (MD) and primary biological aerosol particles (PBAPs) represent an important component of the global atmospheric aerosol. Such particles are important to study, e.g., because they can dominate the aerosol mass-distribution in desert and agricultural regions and can be transported thousands of kilometers while making large contributions to the aerosol optical depth^1,23,36–38^. Both the shortwave and longwave radiative forcing of CMAs remain uncertain to varying degrees^19,28,39,40^. It is even unclear whether CMA particles, MD in particular, have a net global heating or cooling effect on the atmosphere^6,19^. An improved understanding for the size and *shapes* of CMA particles could improve the accuracy of remote-sensing retrievals from ground and satellite observations^21,23,32^.

The most suitable technique to study aerosol particles in their native environment is optical light scattering because measurements can be made in a contact-free and rapid way^41^. Since a particle’s scattering pattern depends on its morphology, composition, and orientation, proper interpretation of a measured pattern can be useful for characterization. Unfortunately, no unambiguous relationship between a measured pattern and these particle characteristics is available, a difficulty known as the inverse problem^41,42^. With the exception of spherical particles^41,43,44^, conventional methods have largely failed to solve the inverse problem and confidently characterize arbitrary particles in situ^41^. Indeed, coarse-mode particles are particularly challenging because of their large optical size, complex shape, and inhomogeneous composition^45^.

Here, we overcome limitations due to the inverse problem using in-line digital holography (DH) and show that images of particles can be obtained while still benefiting from the sample-collection-free nature of conventional light-scattering methods. This is achieved with a new field instrument, the holographic aerosol particle imager (HAPI), which attaches to a low-cost unmanned aerial vehicle (UAV) and images CMA particles as they are in the atmosphere. The HAPI instrument is deployed at several locations in the vicinity of Kansas State University (KSU) to investigate aerosol particles emitted from two sources. These include live pollen emitted from a spruce tree (*picea abies*) and mineral dust from agricultural road traffic. These proof-of-principle results demonstrate that CMA particles $>10 \mathrm{\mu m}$ in size are readily imaged in a sensing volume of $\sim 3 {\mathrm{cm}}^{3}$ with enough detail to resolve the size and shape of these large particles.

**Figure 1:**
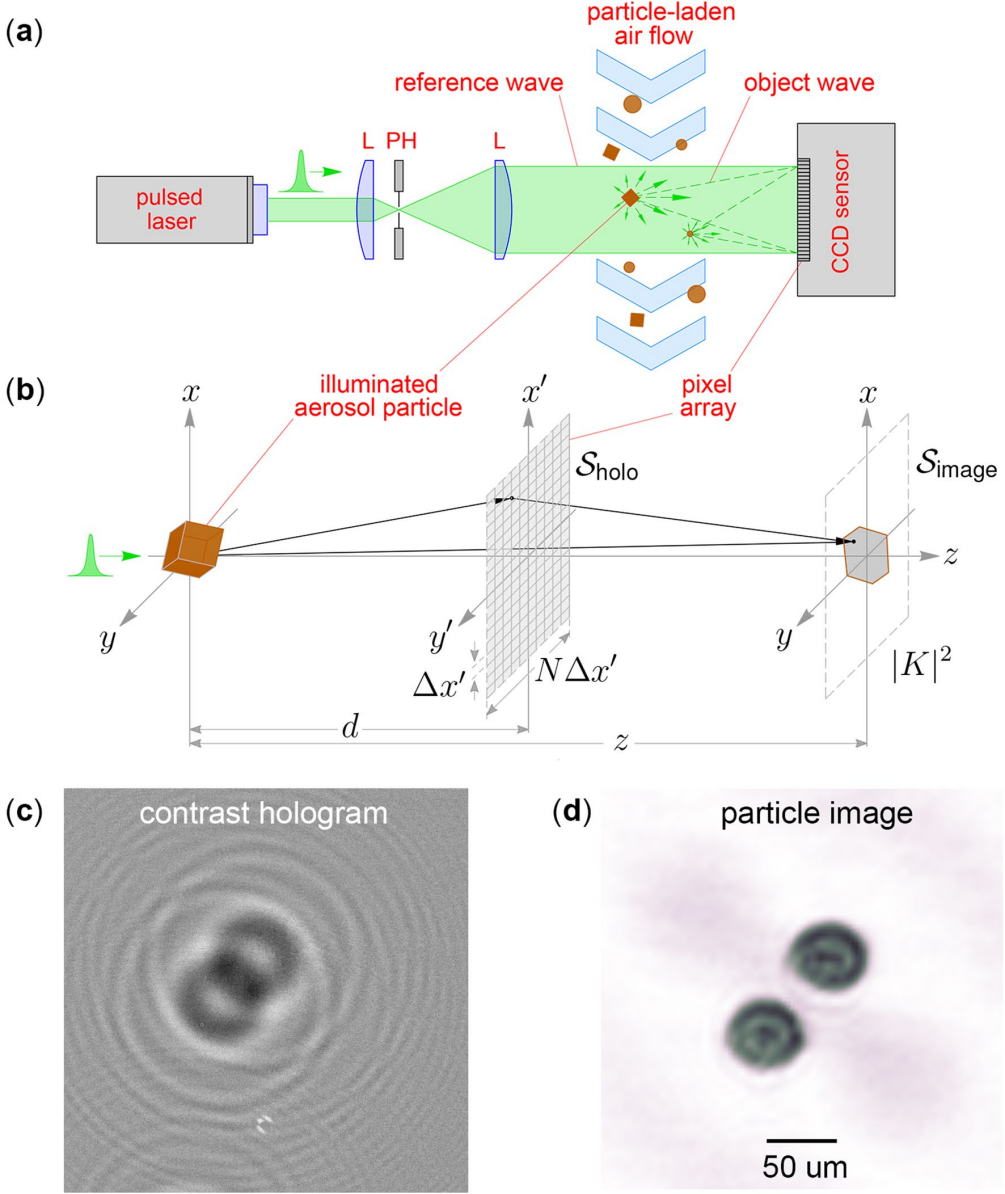
Basic operational principle of digital holographic imaging of aerosol particles. (**a**) A pulsed, expanded laser beam illuminates free-flowing aerosol particles and a CCD sensor records the interference pattern produced by unscattered and particle-scattered light. (**b**) Diagram of the image reconstruction process where the hologram is envisioned as a transmission diffraction grating in the plane ${S}_{\mathrm{holo}}$ that produces an image ${\left|K\right|}^{2}$ in the plane ${S}_{\mathrm{image}}$ through application of diffraction theory (see “Methods”). (**c**,**d**) Example of a contrast hologram ${I}^{\mathrm{con}}$ for an aerosol of spherical particles from^45^ and the particle image obtained from it.

The general imaging principle of in-line DH, which is the simplest configuration suitable for aerosol characterization, is summarized in Fig. 1. In Fig. 1a, a laser beam is expanded by a pinhole spatial filter, improving the beam profile, which then illuminates the surface of a CCD sensor. A flow of aerosol particles traverses the beam at a distance of several centimetres from the sensor. Provided that the particles are much smaller than the beam diameter, the majority of the beam propagates to the sensor unperturbed. This portion is called the reference wave. The small amount of light scattered by the particles received by the sensor constitutes the object wave. These waves interfere, producing an intensity fringe pattern ${I}^{\mathrm{holo}}$ across the sensor, and it is this pattern that constitutes the hologram. By pulsing the laser source, any motion of flowing particles can be frozen, permitting clear fringes to be resolved in the hologram.

Figure 1b depicts the reconstruction process that is applied to the recorded hologram to yield an image. While the details are described in the Methods section, the process conceptually follows that of diffraction. The hologram is viewed as a transmission diffraction grating that resides in the ${S}_{\mathrm{holo}}$ plane such that illuminating it with the beam used to record the hologram will produce a diffracted light-field $K$ in an imaging plane ${S}_{\mathrm{image}}$. Scalar diffraction theory is applied to evaluate computationally this diffraction process, yielding the complex-valued $K$ from the hologram^46,47^. Evaluating ${\left|K\right|}^{2}$ gives an intensity distribution that appears as a silhouette image of the particle. In practice, the process is not applied directly to ${I}^{\mathrm{holo}}$, but rather, to the contrast hologram ${I}^{\mathrm{con}}$, which is produced after subtracting a background measurement, ${I}^{\mathrm{ref}}$. This subtraction step improves the hologram fringe contrast and eventual image. An example of a contrast hologram typical of aerosol particle measurements is shown in Fig. 1c along with the reconstructed image in Fig. 1d. Because of the in-line optical configuration, both a real and virtual image of a particle is produced. When one image is reconstructed with best focus, the other image, the twin, is unfocused and perturbs the focused image. A number of strategies are available to minimize, or even remove, the unfocused twin and that of^48^ is used here to remove the effects of the twin.

The key property of DH is that optical phase information is encoded in the intensity interference pattern measurement. In other words, the function $K$ is complex valued, $K\in {\mathbb{C}}^{2}$, which ultimately accounts for the ability to form the image via scalar diffraction theory^46^. A great variety of useful analysis can be derived from this unique property extending beyond aerosol applications including sub-micron imaging of biological cells^49^.

The unique potential of DH for aerosol characterization is highlighted by the multiple instruments previously developed to study a variety of aerosol properties in different environmental conditions. An early example of a particle-imaging instrument is the cloud particle imager, which employs a diffraction analysis to image high-altitude cloud ice particles^50,51^. Others include the aircraft-mounted HOLODEC instrument for cloud-ice particle imaging^52^, the stationary HOLIMO II instrument^53^ and similar HoloBalloon instrument^54^, a submersible DH imager^55^, a DH cloud imager for cable cars called HoloGondel^56^, a stationary pollen imager^57^, and the ICEMET cloud-ice imager^58^. The HAPI instrument described here has features that make it ideally suited for field research in a cost-effective manner in that it need not be carried by commercial aircraft or a research balloon. The optical design permits a large sensing volume where hologram recording events may be triggered regardless of the direction that particles enter the instrument. This is important as the air flow direction can hamper the aerosol investigations in other instruments. In addition, the design realizes a low size, weight, and power (SWaP), much less than comparable instruments, which enables its operation from inexpensive UAVs.

## Results

**Figure 2:**
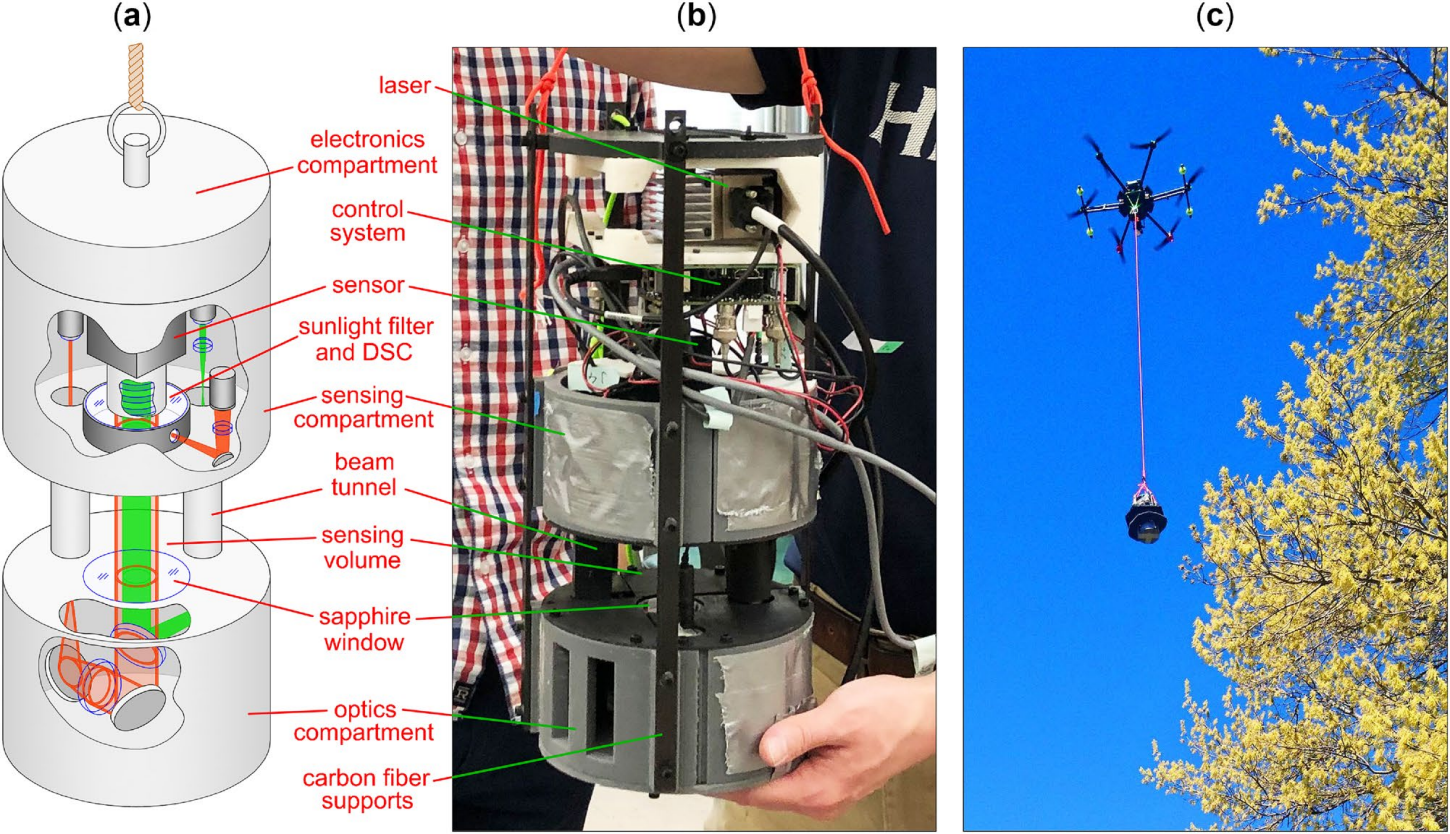
Holographic Aerosol Particle Imager (HAPI). (**a**) Model showing the major components of the instrument, which include the electronics, sensing, and optics compartments. Cutaways from the outer enclosure show the approximate configuration of the elements as labelled (see “Methods”). (**b**) Photograph of the actual instrument with portions of the outer protective casing removed. Leaders identify the location of the components relative to the diagram in (**a**). (**c**) A photograph of the HAPI instrument in-flight during a field trial near a pollinating tree. The black, disk-shaped objects visible on the instrument are sunshades that help limit the intensity of ambient sunlight reaching the sensing volume.

Figure 2a,b present the HAPI instrument, which is described in further detail in “Methods” section. The instrument consists of three stacked cylindrical containers. From top to bottom these include: an electronics compartment where the control and hologram storage systems are contained, a sensing compartment where the CCD sensor and laser sources are housed, and an optics compartment where beams are shaped and combined. A UAV can carry the instrument through the atmosphere via a tether as shown in Fig. 2c, collecting holograms of particles that pass through the sensing volume. The optical system achieving these measurements consists of two beam-paths: a trigger beam and a holography beam. When a particle enters the sensing region, it first encounters a CW annular red-laser beam of wavelength ${\lambda }_{\mathrm{r}}=635$ nm. The particle scatters a small portion of this light, which is then detected by a photomultiplier sensor providing a trigger signal to the electronics system. This trigger queues a pulse from a green laser of wavelength ${\lambda }_{\mathrm{g}}=514$ nm that is adjustable in length between $\tau =10 \mathrm{and }200$ ns. This green beam is approximately one centimeter in diameter and is coaxial with and contained within the red annular trigger beam. An electronic delay in the control system allows time for the particle to pass from the annulus of the trigger beam into the green beam-path by the time the pulse is activated. In this way, it possible to record a digital hologram of all particles in the green beam path (in the sensing volume) and reconstruct the particle images post-measurement.

**Figure 3:**
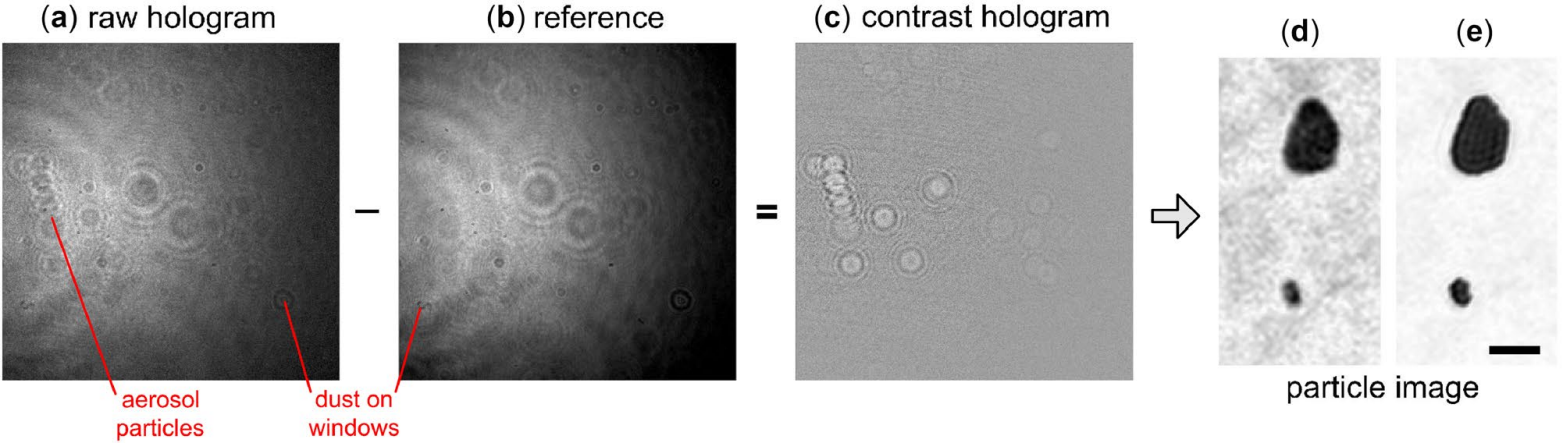
Demonstration of aerosol particle imaging from a contrast hologram. (**a**) Raw hologram ${I}^{\mathrm{holo}}$ obtained from a trigger event during a flight of the HAPI instrument near a pollinating spruce tree. (**b**) Reference measurement ${I}^{\mathrm{ref}}$ of the beam profile without aerosol particles present. The image shows numerous fringe-features due to particles that naturally collect on the windows isolating the internal components of the instrument from the environment. (**c**) Contrast hologram ${I}^{\mathrm{con}}$ showing elimination of most of these unwanted features. (**d**) Reconstructed image of aerosol particles from ${I}^{\mathrm{con}}$ following Eqs. (1) and (2) in “Methods” section. Only a small portion of the full image is shown in (**d**) to highlight the detail of the individual particles. (**e**) Effect of the twin-image removal procedure of^48^ on the image in (**d**) described in “Methods” section. The scale bar in (**e**) is $100 \mathrm{\mu m}$.

While it is possible to reconstruct particle images directly from the measured hologram (see “Methods”), improved images are obtained by performing a background subtraction procedure. This entails readout of the CCD illuminated by a pulse when no particles are present, i.e., it is simply a recording of the undisturbed beam profile. Subtracting this measurement, ${I}^{\mathrm{ref}}$, from a raw hologram ${I}^{\mathrm{holo}}$ with particles present yields a contrast hologram ${I}^{\mathrm{con}}={I}^{\mathrm{holo}}-{I}^{\mathrm{ref}}$. The resulting particle image is improved because stray light and sensor noise largely cancel out in the difference improving the fringe-pattern contrast. Figure 3 shows an example from a flight of the HAPI instrument around an actively pollinating spruce tree. Raw and reference measurements are shown Fig. 3a,b, subtracted to yield ${I}^{\mathrm{con}}$ in Fig. 3c, which is then processed to give an image of aerosol particles Fig. 3d present in the instrument during a trigger event. Figure 3e shows the result of applying the twin-image removal procedure of^48^ to the reconstructed image in Fig. 3d.

Following extensive performance characterization and calibration tests in the laboratory (see “Methods”), the HAPI instrument is flown in proximity to active CMA particle sources at KSU. These include a pollinating spruce tree as PBAP source (see Supplementary Movie S1) and vehicular traffic on a dirt farm road (see Supplementary Movie S2) as an MD source. These trials are referred to as “spruce tree” and “road dust”, respectively. The field trials are conducted midday in clear weather with calm winds to avoid control issues with the UAV and to minimize the swinging motion of the tethered instrument. When flown, the instrument samples the atmosphere over a vertical height of approximately one to 33 m, which roughly corresponds to the physical height of the spruce tree. The duration of the flights varies, but all are under 15 min due to the UAV’s limited battery capacity.

**Figure 4:**
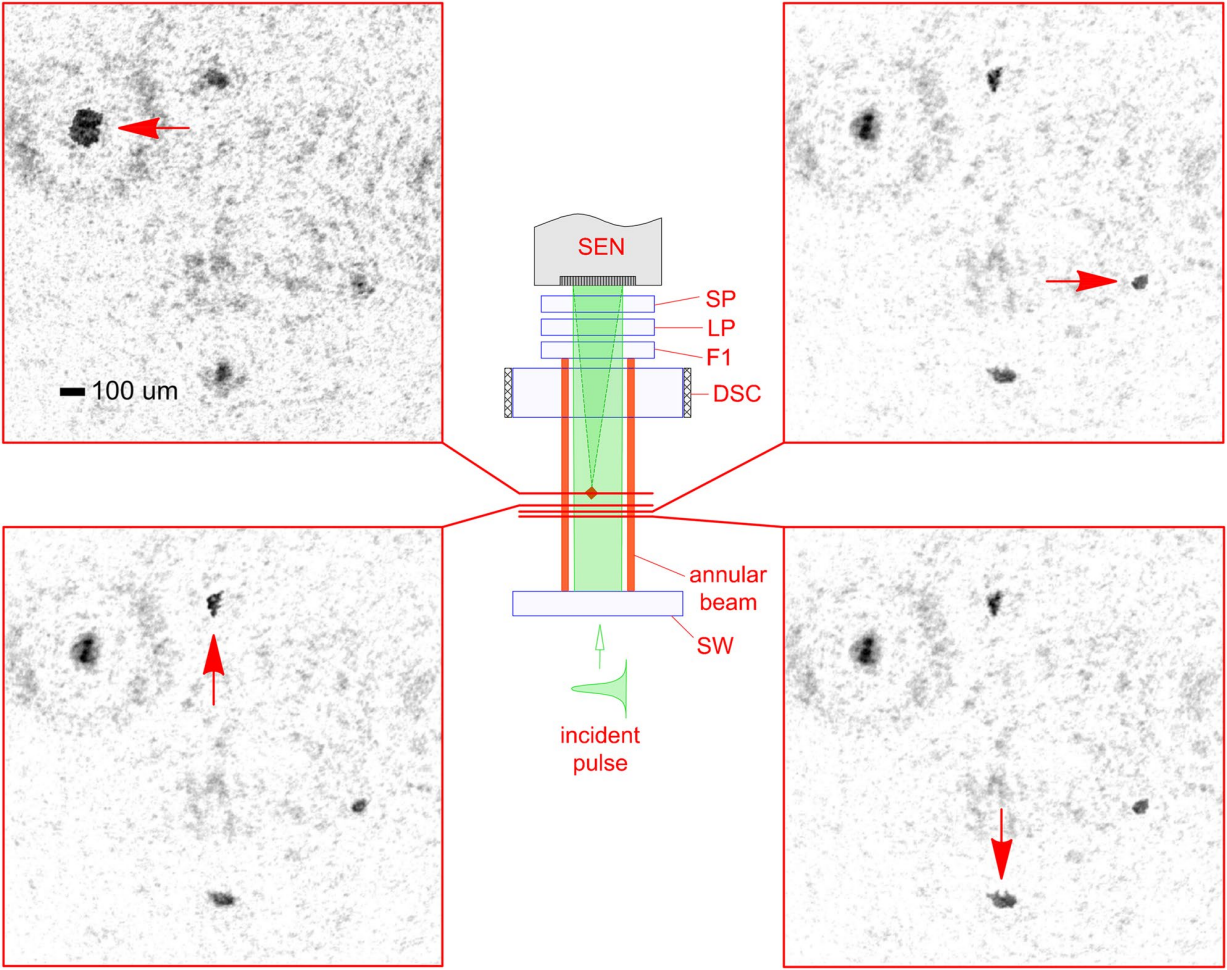
Volume imaging capability of HAPI. Images of aerosol particles reconstructed from a *single* hologram showing particles at different axial locations in the sensing volume as indicated in the central diagram. The field trial is a flight of the HAPI instrument over an agricultural dirt road with active traffic. Red arrows in the images show the particle that is in best focus.

**Figure 5:**
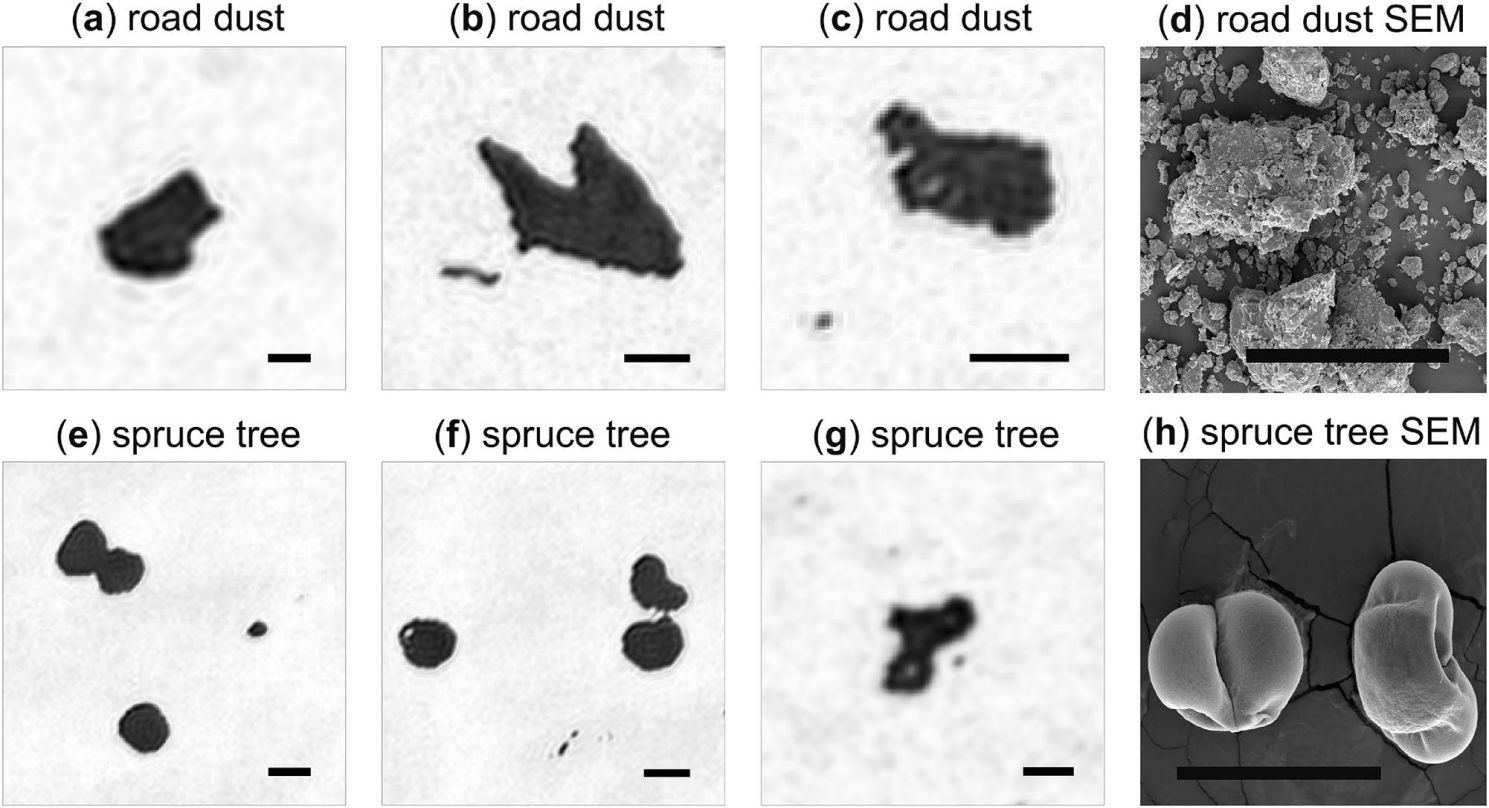
Survey of a variety of suspended atmospheric aerosol particles imaged in situ with the HAPI instrument. (**a**–**c**) Hologram-derived images of aerosol particles observed in the road dust field trial. (**d**) Scanning electron microscope (SEM) image of particles collected from the road dust site. (**e**–**g**) Same as (**a**–**c**) except for aerosol particles observed in the spruce tree trial. (**h**) SEM image of pollen particles collected from the spruce tree. Each image shows a different field of view and all scale bars are 100 $\mathrm{\mu m}$. Qualitative comparison of the aerosol particle images with the SEM images supports an identification of the road dust particles as MD and of the spruce tree particles as pollen grains.

**Figure 6:**
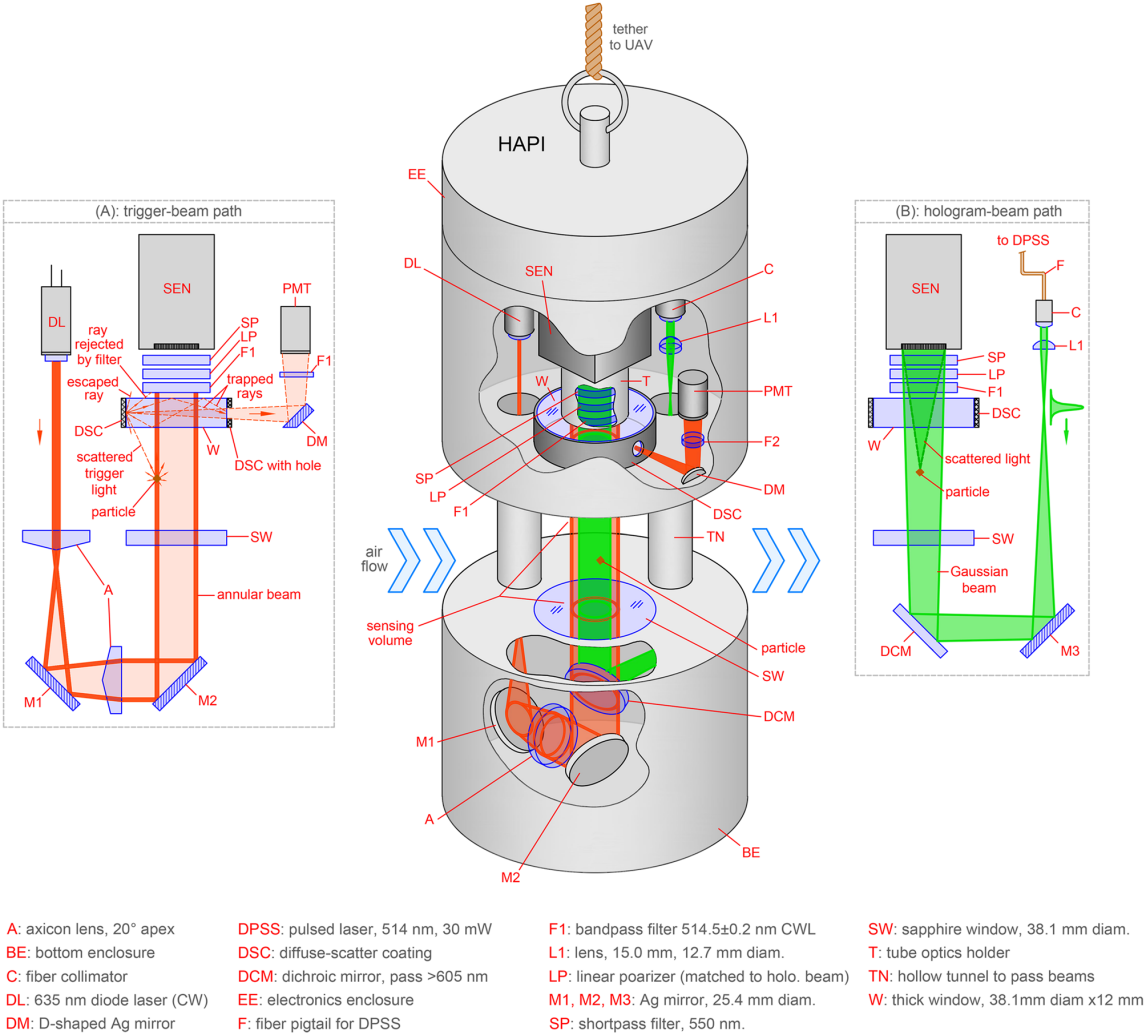
Detailed diagram of the HAPI instrument. (**a**) Layout of the trigger beam path used to sense aerosol particles entering the sensing volume. (**b**) Hologram-forming beam path. These beam paths are oriented perpendicular to each other and the beams overlap co-axially as shown by the 3D diagram of the instrument in the center.

To further highlight the capability of holographic imaging, consider Figs. 4 and 5 where results are presented from flights of the HAPI instrument in the two field trials. In Fig. 4, the aerosol source is traffic on a dirt road (see Supplementary Movie S2) in an agricultural research farm at KSU. When a trigger event occurs and the hologram-forming pulse is emitted, all particles present in the portion of the hologram beam-path in the sensing volume will contribute to the recorded hologram. Thus, in principle, the image reconstruction process can recover images of all of these particles. The sensing volume is approximately $\sim 3 {\mathrm{cm}}^{3}$ and is defined by the cross-sectional area of the hologram beam, approximately one centimeter in diameter, and the distance between the sapphire window (SW) and the thick window (W) used to form trigger signals in Fig. 6 in “Methods”, which is approximately 4 cm. Here, four such particles are presented where their location in the beam differs as shown in relation to the sensing volume in the HAPI model. Due to the field setting and particle size, these particles are likely MD suspended by the mechanical action of the road traffic. Simply adjusting the focus distance parameter $z$ in Eq. (1) below (“Methods”) allows each particle to be brought into focus individually from a *single* hologram, illustrating the significant advantage holographic imaging has over conventional imaging. That is, it would be highly challenging to, e.g., use a long working-distance microscope objective and translate it over distances of centimetres on the time scales necessary to capture focused images of all of these particles before they flow out of the sensing volume.

Next, consider Fig. 5 where further examples of particles observed in the two field trials are presented. Road dust exhibits an irregular shape expected for MD while the spruce tree particles show a less complex, smooth shape. Samples of road dust and pollen from the tree are collected and imaged with a scanning electron microscope (SEM) and shown in Fig. 5d,h. Qualitatively, the particles in the SEM images appear very similar to those imaged by the HAPI instrument, which would support the identification of the particles in Fig. 5a–c as MD and Fig. 5e–g as spruce tree pollen. Indeed, the HAPI-imaged pollen particles in Fig. 5f show hints of the distinctive concavity, or dimple, as those seen in the SEM image and in the literature^59^. Moreover, their size is consistent with the SEM images.

When studies of pollen grains are undertaken, it is common for the grains to be collected from a source, transferred to a laboratory, and then imaged with optical or electron microscopy, e.g., Fig. 5h. In the process, it is likely that the grains become desiccated and crumple, and in such cases, the resulting microscope images will convey an aberrated sense for the true particle shape. This complication is avoided with the HAPI instrument because the holograms of the particles are recorded in situ in the immediate vicinity of the tree as it emits *live* pollen grains. Thus, aside from the limited image resolution, the HAPI images can be regarded as more representative of the true particle shape than one would have from ex situ microscopy of dried pollen. Note that hole-like features can be seen in the particle images in Fig. 5f,g, which are not likely to be real holes through the particles since they are freshly emitted particles. We suspect that these features are artifacts of the twin-image suppression process described in “Methods” where low gray-level features in an image tend to approach the background gray level.

## Discussion

The lack of characterization of aerosols and their loading is one of the greatest uncertainties in climate forcing. While aerosol loadings and their locations can be characterized using lidar techniques, the amount and direction of forcing is also determined by the microphysical properties of the aerosols, such as their size distributions and shape properties. Raman lidar observations, e.g., have demonstrated success in estimating these particle properties with inversion algorithms where spheroidal proxies are used for the aerosol particles, e.g., see^60^. Yet, as Fig. 5 makes clear aerosols of MD are not necessarily well represented by such proxies. Indeed, the very concept of a size distribution is problematic for such particles where more than one characteristic length scale is needed to describe given particle’s size. Determining such particle properties in an unambiguous manner would be best achieved through sampling campaigns or by in situ imaging. We have presented a new instrument that provides the morphological properties, i.e., size and shape, of large aerosol particles in situ using holography and from a commercial-grade UAV. Holography offers distinct advantages over direct-imaging techniques in that there is no set focal plane. A holographic image is brought into focus through data processing, and multiple aerosol particles can be imaged simultaneously from a single hologram. While these images are currently obtained post-processing, it is only a matter of having sufficient computer resources to perform this processing in real time on-site. Doing so, could provide a means of determining the spread of aerosols over an area and tracking them, which can be valuable to study the dynamics of natural and manmade aerosols, like smoke, volcanic ash, pollutants, or even biological agents.

## Methods

### Aerosol particle sensing

Figure 6 shows a detailed diagram of the mechanical and optical design of the HAPI instrument including the two beam-paths discussed above. First consider the beam path in Fig. 6a, which is used to sense the presence of aerosol particles and provide a trigger signal to the instrument’s control system. The optical arrangement is composed of a CW 4.5 mW, ${\lambda }_{\mathrm{r}}=635 \mathrm{nm}$ (red) diode laser module (Thorlabs), which is expanded and formed into a collimated, hollow, annular beam approximately 1.5 cm in diameter by two axicon lenses, each with a $20^\circ$ apex angle. The beam is folded by two mirrors, directed vertically, and then passed through a dichroic mirror (see center diagram in Fig. 6) to the sensing volume. A particle entering the sensing volume from any direction is illuminated by this annular beam, resulting in a small portion of the red light to be scattered as shown. This scattered light is most intense in the forward direction, which is vertical in Fig. 6a, and it then encounters a ~ 1 cm thick silica window (W) with anti-reflection (AR) coating. The side (edge) of the window has the texture of ground glass and is coated with a reflective paint forming a diffuse scattering coating (DSC). A small portion of particle-scattered red light enters the window and strikes the DSC, where it is scattered back into the glass where some becomes trapped via total internal reflection. A small opening in the DSC allows a portion of this trapped light to exit the window where it is reflected to a sensitive micro photomultiplier sensor (PMT, Hamamatsu H12403-01). The PMT is guarded by a narrow-line filter to prevent saturation of the sensor by ambient sunlight or stray laser light. In this way, the DSC window acts as a collector for the weak scattered light from particles entering the sensing volume from any direction*,* and the PMT signals become triggers for the hologram recording process below.

### Hologram recording

Next consider the beam path shown in Fig. 6b, which is used to generate the holographic interference patterns on the CCD sensor. The laser source is a 30 mW diode-pumped solid-state laser of wavelength ${\lambda }_{\mathrm{g}}=514 \mathrm{nm}$, i.e. green, (Coherent OBIS LX FP) with a fiber pigtail mated to a collimator. The beam is passed to a 15 mm focal length positive lens and diverges moderately as it propagates such that it is approximately one centimeter in diameter at the CCD sensor. After being folded by a mirror, the beam is reflected vertically by a longpass dichroic mirror (cutoff 605 nm), and then through a sapphire window (SW) into the sensing volume. The dichroic mirror allows this green beam to be coaligned with the hollow annular trigger beam such that both propagate along the same axis vertically in Fig. 6 through the sensing volume. A particle scattering the red trigger beam and activating the trigger system will continue to travel where it is then illuminated by a pulse of the green beam, which is approximately $D\sim 1$ cm in diameter in this region. Thus, the sensing region is defined as the volume of the (green) hologram-forming beam contained within the annular trigger beam over the distance $\ell \sim 4$ cm between the sapphire window (SW) and triggering window (W) in Fig. 6, which is a volume of ${V}_{\mathrm{sen}}=\pi \ell {D}^{2}/4$ or $\sim 3 {\mathrm{cm}}^{3}$. Scattered and unscattered green light propagate vertically in the figure through the DSC window and then encounter a series of filters. These filters include a linear polarizer, a narrow-line filter with a center wavelength of $514.5 \pm 1\mathrm{ nm}$, and a shortpass filter (cutoff 550 nm). Finally, the light is received by the CCD sensor (FLIR GS3-U3-123S6M-C), located approximately 4 cm from the triggering window, with a $4096\times 3000$ array of $3.45\times 3.45 {\mathrm{\mu m}}^{2}$ pixels and a raw hologram, ${I}^{\mathrm{holo}}$ , is recorded from the green light. The sequence of filters blocks enough of the ambient sunlight and stray trigger-beam light that the CCD sensor is not saturated. We find that the polarizer does not significantly affect the hologram quality as it is polarized in the same direction as the hologram beam.

In order to form the contrast hologram ${I}^{\mathrm{con}}$ discussed in the Introduction, a background measurement, usually called the reference ${I}^{\mathrm{ref}}$, is needed. In the ideal case, the reference represents an exposure of the sensor when no particles are present and consists essentially of the hologram-beam profile. When operating in the outdoor environment however, it is difficult to find a set of conditions that ensure the absence of particles in the sensing volume such that a single sensor-exposure could provide a suitable reference. Moreover, the (small) amount of ambient sunlight reaching the sensor in Fig. 6 can vary slightly during a measurement session. We find that by simply averaging approximately 75 of the raw holograms, ${I}^{\mathrm{holo}}$, resulting from trigger events during a measurement session, we obtain a good substitute for a true reference measurement. The reason for this is because averaging washes-out the fringes due to the particles that flow through the sensing volume, leaving a reference consisting of the hologram-beam profile and fringes from any particles that have affixed to the windows (SW) or (W), and thus, contribute to each ${I}^{\mathrm{holo}}$ in the average. Figure 3 illustrates this process. In Fig. 3a is one of many ${I}^{\mathrm{holo}}$ collected in a measurement session, showing many fringe-structures superposed on the broad Gaussian profile of the hologram beam. Figure 3b shows the reference where the beam profile is clearly present in addition to many of the fringe structures seen in ${I}^{\mathrm{holo}}$, which are particle that have fixed to the window surfaces. A close comparison of Fig. 3a,b reveals some fringe structures not contained in the reference, which are particles flowing through the sensing volume during the triggered exposure of ${I}^{\mathrm{holo}}$. The contrast hologram, ${I}^{\mathrm{con}}= {I}^{\mathrm{holo}}- {I}^{\mathrm{ref}}$, is shown in Fig. 3c where the intensity variation due to the beam profile and fixed particles subtracts out.

### Data processing

Images are reconstructed from contrast holograms ${I}^{\mathrm{con}}$ following scalar diffraction theory as described by^47^. In short, the hologram is viewed as a transmission diffraction grating illuminated by a plane wave. The Rayleigh-Sommerfeld solution to the scalar Helmholtz wave equation provides a description for the diffracted light amplitude, $K$. Referring to Fig. 1b), if the particle-sensor separation $d$ is much greater than ${\lambda }_{\mathrm{g}}$, the Fresnel approximation simplifies the solution to

1 $$K\left(x,y,z\right)={\iint }_{{S}_{\mathrm{holo}}}{I}^{\mathrm{con}}\left({x}^{^{\prime}},{y}^{^{\prime}}\right) h\left(x,{x}^{^{\prime}},y,{y}^{^{\prime}},z\right)\mathrm{ d}{x}^{^{\prime}}\mathrm{d}{y}^{^{\prime}},$$

where $h$ is the impulse response function of free space,

2 $$h\left(x,{x}^{^{\prime}},y,{y}^{^{\prime}},z\right)=\frac{{e}^{\frac{2\pi iz}{{\lambda }_{\mathrm{g}}}}}{i{\lambda }_{\mathrm{g}}z}\mathrm{exp}\left\{\frac{i\pi }{{\lambda }_{\mathrm{g}}z}\left[{\left(x-{x}^{^{\prime}}\right)}^{2}+{\left(y-{y}^{^{\prime}}\right)}^{2}\right]\right\}.$$

In Eq. (1), ${S}_{\mathrm{holo}}$ is the hologram plane, i.e., the sensor, and evaluating ${\left|K\right|}^{2}$ yields a gray-scale silhouette image of the particle in the ${S}_{\mathrm{image}}$ plane by systematically adjusting the parameter $z$ to bring the image into focus^46^. Notice that Eq. (1) is a convolution integral of the hologram with the response function as the kernel. The equation can be efficiently evaluated, e.g., using Mathematica as is done in this work, or through use of fast Fourier transforms with comparable efficiency. Once a focus distance $z$ is found, the unfocused twin image is removed following^48^. The process involves first forming a binarized mask from the focused image in ${S}_{\mathrm{image}}$, backpropagating the mask to ${S}_{\mathrm{holo}}$ via Eq. (1), and then subtracting the resulting complex-valued amplitude from ${I}^{\mathrm{con}}$. This creates a new hologram that is again used in Eq. (1) to produce an updated image in ${S}_{\mathrm{image}}$. The process is iterated until the effects of the twin image vanish, which usually occurs in approximately 20 iterations.

While the image reconstruction procedure above produces high quality images, many holograms are typically recorded in a given field trial and applying this procedure to the recorded data would require a prohibitively large amount of time. Thus, the hologram data from a field trial is preprocessed with a fast image-reconstruction toolkit to identify the holograms most likely to yield quality particle-images. The image reconstructions in this preprocessing stage are performed with a Graphics Processing Unit (GPU) algorithm. Given the large sensing volume, the work is done in two phases. First, the algorithm reconstructs images throughout the full volume in coarse depth-steps along the $z$-axis wherein particles are detected with the autofocus method in^61,62^. For each particle detected, a separate series of reconstructions are run in fine depth-steps to determine values for the focus distances $z$ of the particles in the full sensing volume.

To check that the particle-image length scales and particle locations are accurately obtained by this reconstruction procedure, a $245 \mathrm{\mu m}$ diameter optical fiber is moved along the $z$-axis in the sensing volume in laboratory tests. The approximate locations of the fiber agree with that obtained from its image reconstructions and establish that length scales of this size, at least, are accurately reconstructed. To verify that particles substantially smaller than the fiber can be reliably imaged, we tested HAPI in the laboratory with smaller particles including dried ragweed pollen grains, which are nominally $25 \mathrm{\mu m}$ in size.

A special property of the convolution approach to image reconstruction in DH, i.e., Eq. (1), is that the resolution of the reconstructed image is, in principle, the same as the physical resolution of the sensor array (aside from diffraction limits), i.e., the pixel size^46^. That would suggest that an image resolution as small as $3.45 \mathrm{\mu m}$ is possible. Unfortunately, that is not the case here due to the imperfect cancellation of ambient sunlight and speckle noise, which is the primary factor degrading the resolution. As shown in^46^, speckle noise in the reconstructed image increases with the distance of a particle from the sensor, i.e., its *z*-coordinate (recall Fig. 1), as ${d}_{\mathrm{sp}}=2.44\lambda z/(N\Delta x)$, where ${d}_{\mathrm{sp}}$ is the speckle size and $N\Delta x$ is the size of the sensor array. For the largest *z*-coordinate distances that a particle can obtain in HAPI’s sensing volume, the resolution is thus degraded to $\sim 10 \mathrm{\mu m}$. We thus estimate this value to be the best image resolution for HAPI, which is reasonable given that images of the ragweed pollen grains are well resolved and is a value consistent with our previous work in similar DH experiments in^45^. In future work, it would be straightforward to improve the image resolution by redesigning HAPI to bring the sensor as close as possible to the sensing volume. While we have not yet attempted to do so, a sensor to sensing region distance of 1 cm could enable an image resolution of approximately $1.5 \mathrm{\mu m}$, and laboratory work by^63^ provides some evidence that this is possible.

### Electronic control system

The operational scheme for the electronic control system begins with a circuit that monitors the PMT output voltage for changes of a sufficient magnitude, which correspond to reception of weakly scattered light by particles entering the annular trigger beam. The voltage level required to activate the system is defined by an adjustable threshold ${V}_{\mathrm{th}}$. Once ${V}_{\mathrm{th}}$ is exceeded, by a particle nominally lager than $50 \mathrm{\mu m}$ passing through the annulus of the trigger beam, the electronic shutter of the CCD is activated and a TTL trigger signal is sent to the green (hologram) laser to initiate emission of a pulse. The CCD electronic-shutter then closes and the exposure is written to a Raspberry Pi (Raspberry Pi 3 Model B+) and stored for later analysis. While not done in this work, with sufficient onboard hardware, or data streaming bandwidth to a ground station, the fast image-reconstruction algorithm above could be implemented in near real-time.

Despite the narrow-line filter guarding the PMT, there is always a small amount of ambient sunlight that reaches the PMT, meaning that the sensor is continuously outputting a fluctuating voltage. Particles entering, and thus scattering, the trigger beam add to this fluctuating signal by different amounts depending on their size. Moreover, the gain $G$ of the PMT can be adjusted by a control voltage ${V}_{\mathrm{c}}$, giving a gain that is an exponential function of ${V}_{\mathrm{c}}$, ranging from $G=1-{10}^{5}.$ The system must determine the proper value for ${V}_{\mathrm{c}}$ that yields a gain sufficient to trigger from particles and this is done at the beginning of each measurement event. If the gain is too high, then the system will trigger continuously from the ambient sunlight, and if the gain is too weak, then even the largest particles may not trigger the system. With the instrument placed in the environment where the measurements are to be performed, e.g. on the ground or in a stable hover, a search algorithm finds the appropriate levels for ${V}_{\mathrm{th}}$ and ${V}_{\mathrm{c}}$ that will prevent triggers from the ambient sunlight level while still allowing triggers from particles approximately $50 \mathrm{\mu m}$ and larger. This does not mean, however, that only particles of this size are imaged by the instrument. In practice, a large particle $>50 \mathrm{\mu m}$ will cause a trigger, but a number of far smaller particles will also be in the hologram beam, and thus, are also imaged. Examples of these smaller particles can be seen in Fig. 5b,c,e.

Once a trigger event occurs, the green laser emits a pulse with an adjustable duration between $\tau$ = 10–200 ns. Assuming a relative motion of 1 m/s for the particles, a $\tau =100 \, \mathrm{ns}$ pulse corresponds to a shift in particle position of 100 nm, or approximately one fifth of a wavelength, during the particle illumination. Thus, a clear interference fringe pattern is recorded by the CCD sensor. The CCD exposure time can be adjusted between 1 and 10 ms such that it encompasses the laser pulse. Due to the filters guarding the sensor, the background sunlight contributes negligibly to an exposure even 10 ms in length. Additionally, the laser pulse can be delayed between 1 and 10 ms to allow the particles to travel closer to the center of the hologram beam path.

All data is stored on a MicroSD card in the RPi. Each full-resolution exposure of the CCD generates an 8 MB file, and thus, a 64 GB MicroSD card is used. At sampling rates of approximately 1 Hz, this corresponds to over two hours of continuous measurements, greatly exceeding the typical instrument-flight duration. Alternatively, it is possible to stream data to a laptop via a wireless connection, although, bandwidth limitations make local storage on the MicroSD card preferable for the full resolution data. However, to facilitate real-time monitoring of the data, a thumbnail view is created for each hologram by the RPi that is small enough in file size (under 200 kB) that it is streamed to the operator’s laptop. In this way, the operator is able to tell if useful data is being collected.

### Optomechanics

The core challenge with the mechanical design of the HAPI instrument is to balance the stability requirements of optical control with minimization of the total mass and size of the instrument. Additionally, the two-beam optical design, i.e., Fig. 6a,b, motivates that the beam planes be perpendicular to minimize the overall instrument size, which warrants a three-dimensional design, instead of a two-dimensional single-plane design. The mechanical design can be split into three main components. First are the (planar) beam frames, each of which are support structures for the optical elements shown in Fig. 6a,b containing slots for custom made optical-component holders. Second are the component holders themselves. Third is the exterior casing that fixes the beam frames and electronics in place and attaches to the measurement platform, whether a drone or a static mounting post. The majority of these structural elements, particularly the component holders, are fabricated with a 3D printer (Formlabs, Form 2) to keep the instrument weight low enough that it can be carried by an inexpensive UAV.

Due to separate beam-frames, each beam path can be assembled and aligned independently on a tabletop and then integrated into the outer casing. Keeping the mounts small is key to minimizing the overall size of the instrument and two types are used, static mounts and kinematic mounts. Static mounts provide no adjustment parallel to a given beam-frame and no rotational adjustment of a component but do allow for perpendicular adjustment relative to a frame. Kinematic mounts are essentially static mounts with a separate nested mount that allows for the component to be rotated about all three axes, i.e., pitch, yaw, and roll. Due to their larger size, kinematic mounts are only used for mirrors in the beam paths that are key to ensuring that the beams will overlap properly in the sensing volume and be centered on the CCD face.

Due to limitations of the 3D printer, much of the outer casing must be printed in pieces and then assembled via screws to form the final structure. As a benefit, this means that pieces of the casing can be removed independently to provide access to the internal optics without having to disassemble the entire instrument. This is convenient for purposes such as re-aligning beams and cleaning optics between field trials. The assembled HAPI instrument is composed of three cylindrical compartments as shown in Fig. 2; including the electronics compartment, sensing compartment, and optics compartment. The compartments are fixed together with external carbon fiber strips, visible in Fig. 2b, and spacing between them is sealed by foil and tape for protection against sunlight and dust. Black foam disks are mounted around the circumference of the instrument on the top and bottom ends of the sensing volume to serve as a sunshade (see Fig. 2c).

### Size, weight and power (SWaP)

The HAPI instrument is cylindrical in form, approximately 20 cm in diameter and 42 cm in length. A tether attached to the carbon-fiber supports is used to suspend it from a UAV as shown in Fig. 2c. The tether is long enough that the instrument can be placed in upright orientation a few meters from the UAV before liftoff and minimizes mechanical vibration of the instrument due to the UAV rotors when aloft (see Supplementary Movie S1). Upon landing, the tether also allows the instrument to be set on the ground in an upright orientation with the UAV landing nearby.

The UAV used is a DJI Matrice 600 Pro multirotor drone (M600), which offered a favorable compromise between payload capacity and cost. With high-end manufacturer batteries (TB48S), the M600 can lift 6 kg of payload. The lighter the payload, the longer the flight time and the easier the UAV is to control. Fully assembled including batteries, the HAPI instrument is approximately 3.3 kg, well under the payload weight limit. A deadweight payload of 5 kg was suspended via the tether from the UAV and flight tests were conducted to establish controllability of the UAV before trials were conducted with the instrument.

For electrical power, the UAV uses its own batteries for flight while the HAPI instrument is powered by additional batteries. At the maximum payload mass, the flight time of the M600 is roughly 15 min, and approximately 35 min with no payload. Thus, the power consumption as well as the battery capacity would have to be balanced to provide for at least approximately 15 min but would not need to exceed 35 min. The HAPI batteries feed a voltage regulator, which then supplies the control electronics and lasers of the instrument. Given that the instrument’s highest voltage requirement is 12 VDC at a maximum of 4 A, two parallel-connected 3-cell lithium polymer batteries (Turnigy 2,200 mAh) are used, supplying approximately 22.2 VDC that is downregulated to 12 VDC by the regulator. With a power consumption of approximately 22.65 W for the HAPI instrument, this provides over two hours of operational time, greatly exceeding the minimum requirement and eliminating the need to switch batteries after every flight.

## Supplementary information

Supplementary Video S1

Supplementary Video S2

Supplementary Video Legends

## Data Availability

All data needed to evaluate the conclusions of this work are included in the manuscript and the Supplementary Material.

## Code availability

Additional data, including code, may be obtained upon request from M.J.B.
