# Supplementary material for: Imaging atmospheric aerosol particles from a UAV with digital holography
Source: Sci Rep. 2020 Sep 30;10:16085. doi: 10.1038/s41598-020-72411-x (PMC7528099; doi:10.1038/s41598-020-72411-x)
Supplement: Supplementary file 3 — Supplementary Video Legends [file 41598_2020_72411_MOESM3_ESM.docx]

Movie S1. Video showing portions of the spruce tree field-trial.

Movie S2. Video showing portions of the road dust field-trial.
